# Complete Genome Sequence of *Lactobacillus plantarum* Oregon-R-modENCODE Strain BDGP2 Isolated from *Drosophila melanogaster* Gut

**DOI:** 10.1128/genomeA.01155-17

**Published:** 2017-10-12

**Authors:** Kenneth H. Wan, Charles Yu, Soo Park, Ann S. Hammonds, Benjamin W. Booth, Susan E. Celniker

**Affiliations:** Biological Systems and Engineering Division, Lawrence Berkeley National Laboratory, Berkeley, California, USA

## Abstract

*Lactobacillus plantarum* Oregon-R-modENCODE strain BDGP2 was isolated from the gut of *Drosophila melanogaster* for functional host microbial interaction studies. The complete genome comprised a single circular genome of 3,407,160 bp, with a G+C content of 44%, and four plasmids.

## GENOME ANNOUNCEMENT

*Lactobacillus plantarum* increases amino acid metabolism and promotes larval growth in *Drosophila melanogaster* under nutrient-scarce conditions (1). Previously published *L. plantarum* strains include one derived from a laboratory fly strain (KP) and one from wild-caught flies (DF) (2). Here, we report the genome sequence of a strain of *L. plantarum*, Oregon-R-modENCODE strain BDGP2 associated with a sequenced *D. melanogaster* host, to facilitate mechanistic studies of host-microbiome interactions.

*L. plantarum* Oregon-R-modENCODE strain BDGP2 was isolated from a fecal swab. Bacteria were streaked onto Difco de Man, Rogosa, and Sharpe (MRS) agar (3) agar (BD catalog number 288210) plates, single colonies were amplified, and an aliquot was used for 16S V1 and V4 PCR (4) and sequence identification (5). DNA was isolated (6), and whole-genome sequencing was performed by the National Center for Genome Resources (NCGR), Santa Fe, New Mexico, USA, using Pacific Biosciences (PacBio, Menlo Park, CA, USA) long-read sequencing on the RS II instrument (7). A single-molecule real-time (SMRT) cell library was constructed with 5 to 10 µg of DNA using the PacBio 20-kb protocol and sequenced on one SMRT cell using P6 polymerase and C4 chemistry with 6-h movie times. Sequencing yielded 82,902 reads with a filtered mean read length of 11,874 bp, totaling 984,453,362 bp (>200-fold coverage of the chromosome and plasmids). The files generated by the PacBio instrument were used for a *de novo* assembly constructed using the HGAP2 protocol from SMRT Analysis version 2.0 (8, 9). The final contigs were manually trimmed and reviewed to produce a single circular chromosome and four plasmids. Annotations of protein-coding open reading frames and noncoding RNAs were predicted using the Rapid Annotation of microbial genomes using Subsystems Technology tool (10) and the GenBank annotation pipeline (11).

The chromosomal genome annotation predicts 3,148 protein-coding genes, 131 pseudogenes, 5 rRNA operons, a single 5S rRNA, and 85 tRNAs (78 with canonical anticodon triplets that base pair with codons for amino acids, 1 suppressor tRNA [tRNA-Sup-TTA], 1 selenocysteine-specific tRNA [tRNA-SeC-TCA], 1 undetermined tRNA [tRNA-OTHER], and 4 pseudo-tRNA genes). Of the 3,148 protein-coding genes, 328 are contained within candidate prophages. Like other *L. plantarum* strains, ours contains integrated likely prophages (Phage_Finder; Omic Tools). There are seven copies, six ranging in size from 36,225 to 46,235 bp and in sequence similarity from 45 to 70% and a partial copy of only 13,544 kb. Together they constitute 7.5% of the genome. Two cornerstone proteins highly conserved in prophages are the large terminase subunit and the portal protein (12). These candidate prophages, including the partial copy, encode a large terminase subunit protein, and all but one of our candidate prophages encode a portal protein. In addition, the genome contains four plasmids, pLtBDGP2A (61,448 bp), pLtBDGP2B (58,689 bp), pLtBDGP2C (41,234 bp), and pLtBDGP2D (13,055 bp). Plasmids pLtBDGP2B and pLtBDGP2C contain candidate genes for the plasmid replication initiation proteins RepE and RepA, respectively. Plasmids pLtBDGP2A and pLtBDGP2D show sequence similarity to *L. plantarum* strain DF plasmid unnamed1 (GenBank accession number CP013754) and *L. plantarum* strain TMW 1.277 (palm wine isolate) plasmid pL1277-4 (accession number CP017367), respectively.

### Accession number(s).

The complete genome sequences of the chromosome and four plasmids of *L. plantarum* Oregon-R-modENCODE strain BDGP2 have been deposited in GenBank under the accession numbers CP023174 (chromosome) and CP023175 (pLtBDGP2A), CP023176 (pLtBDGP2B), CP023177 (pLtBDGP2C), and CP023178 (pLtBDGP2D) (plasmids).
